# Prioritizing homelessness in emergency medicine education: A concept paper

**DOI:** 10.1002/aet2.10753

**Published:** 2022-06-23

**Authors:** Benedict C. Del Buono, Bisan A. Salhi, Alexis E. Kimmel, Sally A. Santen, Kelli L. Jarrell, Melissa H. White, Christopher K. Brown, Joel L. Moll

**Affiliations:** ^1^ Department of Emergency Medicine Virginia Commonwealth University School of Medicine Richmond Virginia USA; ^2^ Department of Emergency Medicine Emory University School of Medicine Atlanta Georgia USA; ^3^ Department of Emergency Medicine University of Cincinnati College of Medicine Cincinnati Ohio USA; ^4^ Virginia Commonwealth University School of Medicine Professor, Emergency Medicine and Medical Education University of Cincinnati College of Medicine Cincinnati Ohio USA

## Abstract

Patients experiencing homelessness visit the emergency department (ED) often and have worse clinical outcomes. Caring for this patient population is complex, challenging, and resource‐intensive. Emergency medicine (EM) education is lacking in formal curricula on the topic of homelessness, despite benefits for resident morale and patient care. Our goals were to identify a gap in EM education and training of the intersection of housing and health and propose educational topics and teaching methods to be included in residency curricula. Methodology was based on the development of a didactic session at the 2021 SAEM Annual Meeting. A needs assessment was performed through a review of medical education literature, a national survey of EM residency curricula, the individual curricula utilized by respective team members, and perspective from the team's own individual experiences with teaching about homelessness. Topics presented were chosen through discussion between the authors and determined to be common and relevant and cover a broad spectrum of content. The four presented topics included the intersection of COVID‐19 and housing, the impact of LGBTQIA+ status on homelessness, housing status related to health system utilization and health outcomes, and housing inequity as a means of perpetuating structural racism. Suggestions for education of these topics included case‐based learning, journal clubs, simulation, collaboration with social work, quality improvement projects, and engagement with community leaders. The ED is uniquely positioned to encounter the impacts of homelessness on health. Emergency physicians should be prepared to effectively care for these patients with complex social needs. Structured learning on this topic would benefit EM resident growth and lead to better patient care through improved screening, recognition of risk factors, and use of social resources.

## INTRODUCTION

Patients experiencing homelessness comprise a common and vulnerable population in emergency medicine (EM). According to the National Hospital Ambulatory Medical Care Survey, in 2017 there were an estimated 990,000 emergency department (ED) visits by individuals experiencing homelessness.^1^ Compared to patients who are housed, individuals experiencing homelessness have increased mortality;^2^, ^3^, ^4^ visit the ED eight times more often;^5^ receive unequal care;^2^ and have higher infectious, mental health, cardiovascular, and respiratory disease prevalence.^6^ Caring for this patient population is complex, challenging, resource‐intensive, and a certainty for emergency physicians throughout their careers.^7^

Despite the development of frameworks that incorporate social determinants of health (SDoH) into medical education and training and recent calls for the inclusion of homelessness in EM residency education,^8^, ^9^, ^10^, ^11^, ^12^, ^13^, ^14^, ^15^ EM lacks any formal or proposed curricula on homelessness. There are, however, qualitative assessments^16^ and surveys^17^ on the resident experience of caring for this patient population. They demonstrated that EM residents learned unique aspects of caring for patients experiencing homelessness through experience and informal teaching rather than a formal curriculum. Residents, however, described feelings of burnout when working with this population and “learning from the misses,” prompting the authors to suggest the “need for improved education to prevent poor outcomes among patients who are homeless.”^16^ Informal teaching and individual experiences are often subject to misinterpretation and bias, thereby questioning the quality of an informal curriculum. A structured and evidence‐based curriculum would likely be an advancement from what education, if any, is available to many EM residents.

Formal education and training on the topic of homelessness can positively affect physician attitudes and patient outcomes. Discrimination related to homelessness and poverty in health care settings has been associated with more frequent ED visits, a greater severity of lifetime substance abuse, and mental health problems.^18^ Reinforcement of negative cognitive biases by physicians has been speculated to lead to missing treatable pathologies.^12^ In multiple studies, dedicated training of physicians on homelessness and the connection between social factors and health reframed how participants viewed patients clinically, improved confidence in recognizing social barriers to health, improved attitudes toward homelessness, and made participants feel more connected to original motivations for entering the health professions.^17^, ^19^, ^20^, ^21^ This connection between education, provider morale, and patient care offers a glimpse into the benefits of further developing EM education and training on homelessness.

Homelessness has a significant presence in the ED; however, there is a lack of literature and formal curricula in EM education dedicated to training physicians to care for this patient population. Further development and sharing of curricula dedicated to this topic is important in fulfilling standards set forth by the 2019 Model of the Clinical Practice of Emergency Medicine (EM Model)^22^ and is likely to improve physician morale and clinical outcomes. In this concept paper, we perform a needs assessment, seek to build curricular content through four key topics pertinent to homelessness and EM, and propose educational modalities for a structured curriculum.

## METHODOLOGY AND NEEDS ASSESSMENT

Methods were based on the development of a didactic session titled “Prioritizing Homelessness in Emergency Medicine Education” presented at the 2021 Society for Academic Emergency Medicine (SAEM) annual meeting. The authors collectively had extensive experience in EM residency education and social EM and included experts from multiple institutions. A needs assessment was performed through several sources to better delineate the landscape of homelessness in EM education and establish content for discussion.

### Literature review

The didactic and this concept paper were supported by a literature review to provide background information about the complex and widespread nature of the topic. In consultation with a health sciences librarian, a series of literature searches were conducted in Medline/PubMed and MedEdPORTAL. Terminology focused on homelessness and SDoH combined with the concepts of education and training, attitudes of physicians, and EM as a setting and specialty.

### National survey

An institutional review board–approved needs assessment survey was distributed through the Council of Residency Directors in Emergency Medicine (CORD) email listserv, which includes members from all EM residency programs in the United States. Survey questions focused on the prevalence of homelessness in curricula, including dedicated time, preferred time, educational modalities, and barriers to implementation (Appendix 1). For response process validity, the survey was reviewed by representatives from two EM programs prior to its distribution, including faculty members with extensive experience in EM education and EM residents. Eligible participants were determined using the Accreditation Council for Graduate Medical Education (ACGME) program database.^23^

**FIGURE 1:**
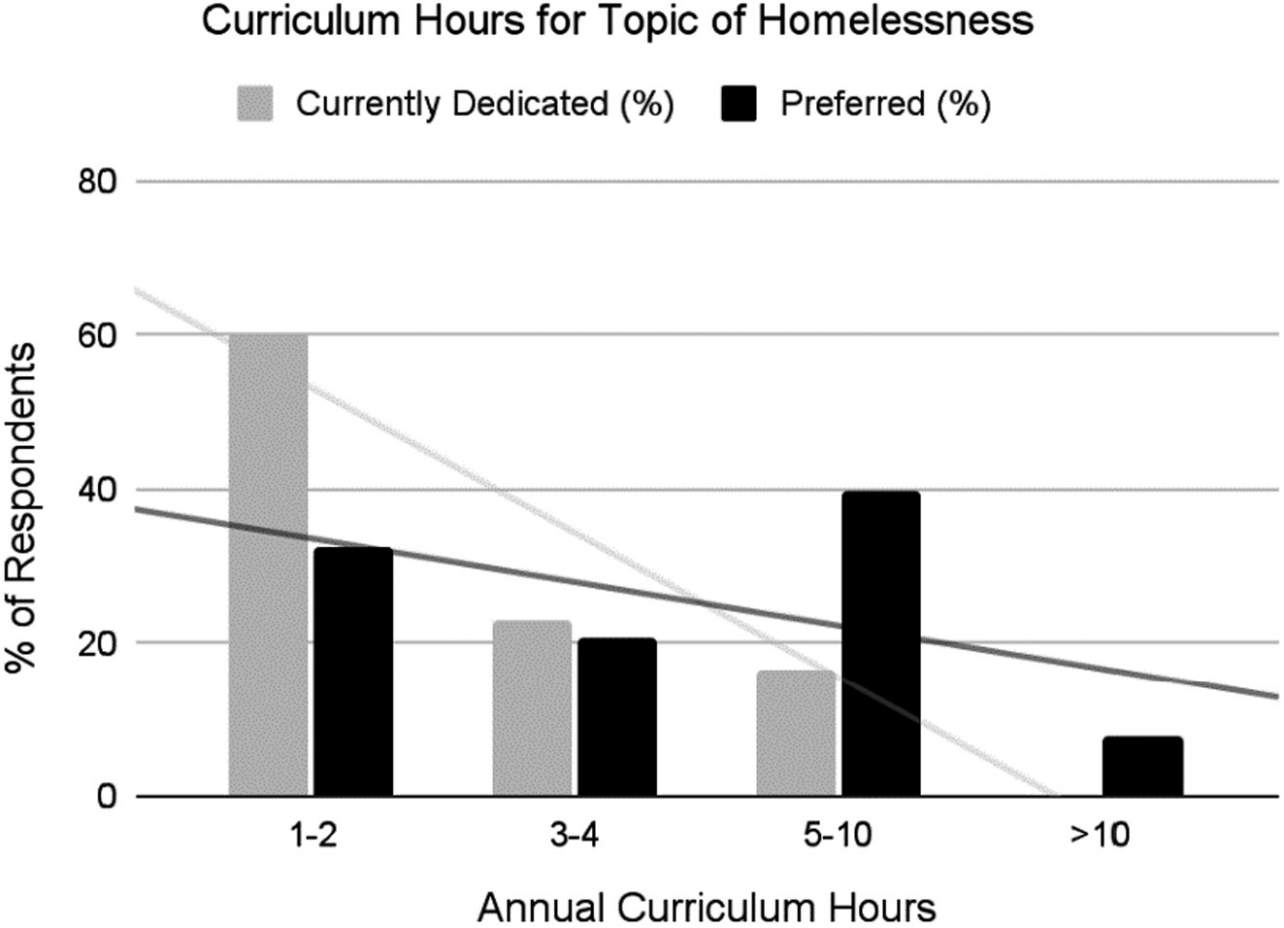
Curriculum hours for topic of homelessness

Fifty‐three programs participated for a response rate of 21.9%. Although the participants composed a diverse group from mostly urban (66.0%) and suburban (32.1%) programs representing 22 different states in all regions of the United States, the low response rate limits generalizability of the data. Most programs reported a “somewhat heavy” or “heavy” exposure to patients experiencing homelessness (69.5%), whereas only 7.5% described their exposure as “minimal.” Forty‐eight programs (90.6%) addressed homelessness in their curricula. The most popular educational methods were collaboration with social work (30/48, 62.5%), dedicated didactics (21/48, 43.8%), and incorporation of the topic into medical lectures and cases (21/48, 43.8%). Of these programs, 29 (60.4%) dedicated 1–2 h on the topic annually, 11 (22.9%) dedicated 3–4 h, and eight (16.7%) dedicated 5–10 h on the topic. Overall, participants desired that more curricular hours per year be dedicated to homelessness, while all participants responded that this topic should be included in their curricula. Seventeen programs (32.1%) preferred 1–2 h, 11 (20.7%) preferred 3–4 h, 21 (39.6%) preferred 5–10 h, and 4 (7.6%) preferred more than 10 h (Figure 1). There was a clear gap between the amount of time that is currently spent on this topic (median 2 h, range 0–10 h) and the amount of time that is preferred (4 h, range 1–26 h). This preferred time, along with the reported high exposure to homelessness among respondents, suggests that about 4 curriculum hours per year should be devoted to this topic. The reported median of 2 h per year with 64.2% of programs spending 2 h or less on homelessness suggested a small presence of curricula nationwide. Lack of interest by programs was not a common barrier to including this topic in education (11.3%). Instead, the availability of time (75.5%) and content expertise among faculty (45.3%) were more commonly reported barriers.

### Goals and objectives for SAEM didactic session

The final component of the needs assessment was to determine goals and objectives for the SAEM didactic session. Individual curricula utilized by respective team members and perspectives from the team's own individual experiences with teaching about SDoH and homelessness were considered. There was noted to be significant overlap in topics and objectives; however, the programs clearly operated in different contexts, indicating generalizability of proposed curriculum content across the programs. The team also informally consulted other EM residency programs to extend the group's understanding and approach to teaching about homelessness. Concepts relevant to SDoH were triangulated with the 2019 EM Model and therefore considered significant for inclusion in residency curricula. Based on the needs assessment, we determined that the goals and objectives were to educate faculty and residents on the importance of homelessness as a SDoH, advocate for incorporation of the topic into EM education, and propose specific content and teaching modalities to be included in curricula. The objectives were to provide attendees support and methods to bring homelessness into their curricula through different approaches.

### Determination of four key topics

This concept paper presents four key topics to meet the objectives of EM resident ability to understand and address these issues in patient care. These four topics were chosen through the needs assessment and discussion between the authors and determined to be common in the literature review, currently relevant, and cover a broad spectrum of content. They are well aligned with the goals and experience of the authors’ programs and have a strong presence in the SAEM and American College of Emergency Physicians (ACEP) Social EM membership sections.^24^, ^25^

## KEY TOPICS AND TEACHING METHODS FOR HOMELESSNESS IN EM

We present four topics within homelessness in EM that demonstrate the intersection of housing and health: COVID‐19, LGBTQIA+ (lesbian, gay, bisexual, transgender, queer or questioning, intersex, asexual, and plus), health system utilization and health outcomes, and structural racism (Table 1). Although considered important, these four topics are not intended to represent the complete content of a curriculum. Table 2 is a comprehensive list of educational topics based on the literature review, the Office of Disease Prevention and Health Promotion, and the SAEM and ACEP social EM sections.^24^, ^25^, ^26^, ^27^ Each presented topic also includes guidance and suggestions for education. The choice of teaching method should ideally be aligned with a specific learning objective. A summary of suggested education modalities related to these topics, along with additional teaching methods, is presented in Table 3. Regardless of chosen methods, personal interaction with the material through discussion and active learning will likely be most effective.

**TABLE 1:** Key concepts

Curriculum topic	Key concepts
COVID‐19	Homelessness is a risk factor for COVID‐19 infection. Eviction and utility moratoria during the pandemic had a significant reduction of COVID‐19 infections and death, highlighting the role of housing in preventing the spread of infectious disease.
LGBTQIA+	LGBTQIA+ individuals disproportionately experience homelessness and are more susceptible to negative health implications, including higher rates of substance abuse, mental illness, sexual and physical abuse, risky behaviors, survival sex, and HIV.
Health system utilization and health outcomes	Homelessness is associated with a higher mortality compared to the general population. Stable housing decreases preventable ED visits and hospitalizations and has been shown to improve HIV outcomes.
Structural racism	Inequitable housing opportunities are driven by structural racism. Poor living conditions among racial minorities are associated with higher ED visits for asthma exacerbations and worse outcomes.

Abbreviations: COVID‐19, coronavirus disease‐19; HIV, human immunodeficiency virus; LGBTQIA+, lesbian, gay, bisexual, transgender, queer or questioning, intersex, asexual, and plus.

**TABLE 2:** Comprehensive list of curriculum educational topics

Educational topic related to homelessness
Access to education
Addiction to drugs and alcohol
Adverse childhood experiences
Economic stability
Exposure to violence
Health outcomes
Health policy
Health system utilization
Immigration and documentation status
Incarceration
Infectious diseases
LGBTQIA+
Mental health
Sexual violence and human trafficking
Structural racism

Abbreviation: LGBTQIA+, lesbian, gay, bisexual, transgender, queer or questioning, intersex, asexual, and plus.

**TABLE 3:** Suggestions for education and teaching methods

Curriculum topic	Suggestions for education
COVID‐19	Case‐based learning and journal club sessions that address disparities of COVID‐19 due to socioeconomic status.
LGBTQIA+	Simulated clinical encounters that challenge residents to ask patients how they identify, screen for homelessness, and facilitate a safe discharge plan.
Health system utilization and health outcomes	Collaboration with social work and quality improvement projects. Morbidity and mortality conference to connect homelessness with clinical outcomes.
Housing inequity and structural racism	Invite community leaders who advocate for racial justice to speak at residency conference.

Education setting	Teaching methods
Didactics	Case studies, lectures, video‐based demonstrations, audience response, muddiest point.
Small group	Concept maps, open discussion, simulation, journal club, buzz groups, think‐pair‐share, problem‐based learning, role‐playing, debates.
Individual	Narrative writing and reflections.
Panel	Patients, allied health providers (i.e., social work), community advocates, content experts.
Action	ED‐based quality improvement projects, community‐based service learning, advocacy planning.

Abbreviations: COVID‐19, coronavirus disease‐19; HIV, human immunodeficiency virus; LGBTQIA+, lesbian, gay, bisexual, transgender, queer or questioning, intersex, asexual, and plus.

### COVID‐19

The COVID‐19 pandemic has exacerbated the inequities of homelessness and been revelatory of the connection between housing and health. Individuals experiencing homelessness have an increased risk of contracting COVID‐19, likely given a high prevalence of medical risk factors and difficulty adhering to public health directives, such as social distancing.^6^, ^28^, ^29^ The cessation of housing evictions during the pandemic has made this clear. Jowers et al.^30^ studied the impact of evictions and utility disconnections on COVID‐19 infections, finding that eviction moratoria reduced COVID‐19 infections by 3.8% and deaths by 11%. Similarly, utility disconnection moratoria reduced COVID‐19 infections by 4.4% and mortality by 7.4%. The authors further estimated that nationwide eviction moratoria could have reduced COVID‐19 infections by 14.2% and deaths by 40.7%, while moratoria on utility disconnections could have reduced infection and deaths by 8.7% and 14.8%, respectively. These data highlight homelessness as a risk factor for contagious infectious disease and suggests that supportive housing plays a significant role in infectious disease prevention in this group.

As the COVID‐19 pandemic continues to play a pivotal role in ED care, special attention must be paid to individuals experiencing homelessness. When caring for these patients, it is important that residents inquire about housing status and the ability to safely quarantine, and advocate for safe discharge to housing. Emergency physicians should understand the increased risk that homelessness has on the spread and mortality of infectious disease. This topic could be integrated into lectures and cased‐based learning on COVID‐19, highlighting how homelessness impairs the ability to perform supportive care and properly quarantine. Journal club can be an effective modality to learn from recent publications on disparities of COVID‐19 related to socioeconomic status. In addition to the references above, relevant articles include a case report and public health approach to infection control^31^ and the association of housing eviction and public utility moratoria with decreased COVID‐19 incidence and mortality.^32^, ^33^

### LGBTQIA+

Persons who identify as LGBTQIA+ are disproportionately affected by homelessness. Estimated data indicate that 20%–40% of LGBTQIA+ individuals experience homelessness, while the prevalence of LGBTQIA+ as a subgroup of the total population is around 5%–10%.^34^ Youth are particularly at risk, as disclosure of their identity can lead to the exacerbation of previous family conflicts, being forced to leave their home, or choosing to leave due to an intolerable environment.^35^ Many conditions with clear health implications commonly associated with homelessness also disproportionately affect LGBTQIA+ individuals compared to non‐LGBTQIA+ individuals. These include higher rates of substance abuse, mental illness, sexual and physical abuse, risky behaviors, survival sex, and human immunodeficiency virus (HIV).^36^, ^37^, ^38^ Transgender individuals experiencing homelessness are often impacted by these problems to an even greater degree.^34^

Disposition of LGBTQIA+ patients from the ED requires additional consideration. Despite the overrepresentation of LGBTQIA+ persons among the total of individuals experiencing homelessness, personnel and resources do not always address the needs of LGBTQIA+ persons.^39^ In addition, consideration of a safe discharge is critically important for marginalized populations, especially youth and advanced age who might be subject to abuse. Simulated clinical encounters can be an effective opportunity to build these skills. Glick et al.^40^ developed a single encounter simulation case that challenged internal medicine residents to recognize and screen for homelessness, adjust their clinical management based on housing status, and effectively refer to social work. Participants who had previously participated in a rotation on health care and homelessness were more likely to successfully manage diabetes control and refer the patient to a social worker. In a simulated case focused on LGBTQIA+, residents should be expected to ask the patient how they identify and screen for homelessness and subsequently incorporate these factors into their assessment and plan. Initiating a safe discharge and connection with necessary social resources can serve as important critical actions.

### Health system utilization and health outcomes

Housing status is intimately tied to health system utilization and health outcomes. Supportive housing programs throughout the United States have shown to decrease ED visits and prevent hospitalizations for patients experiencing homelessness. A randomized controlled trial reported a reduction of 29% in hospitalizations, 29% in hospital days, and 24% in ED visits when patients experiencing homelessness were provided ED case management services and transitional housing versus usual ED discharge planning with no continued support.^41^ In 2018, Lim et al.^42^ demonstrated that a supportive housing program in New York City resulted in 87% of participants remaining housed 2 years later. This group was 40% less likely to make preventable ED visits when compared to a group of unstably housed individuals. Supportive housing has also been demonstrated to improve health outcomes in this patient population. HIV is more common in patients experiencing homelessness^43^ and associated with a lower likelihood of adherence to antiretroviral treatment plans.^44^ Stable housing has been associated with better adherence to HIV treatment regimens^45^ and increased survival with intact immunity and undetectable viral loads.^46^

Stable housing decreases preventable ED visits and hospitalizations and improves health outcomes, particularly in those diagnosed with HIV. Morbidity and mortality conference is an opportunity to portray how overlooking housing status in management plans can lead to adverse clinical outcomes. Residents should be encouraged to identify how homelessness and other SDoH affect risk factors for disease, medical decision making, and safe discharge. Housing and food insecurity have also been described as motivators for ED visits^47^ and led to increased utilization of the ED for preventive care.^48^ Physicians should ask patients about housing status as part of their social history, and it should be routinely considered in treatment, disposition, and follow‐up plans.^49^ Collaboration with social work may be particularly valuable in this effort. A social worker and EM resident training module on Screening, Brief intervention, and Referral to Treatment (SBIRT) methodology was successfully developed to be utilized in the ED.^50^ A program in New Orleans trained medical students to effectively coordinate discharge planning and referral support between physicians and social workers for patients experiencing homelessness.^51^ Quality improvement projects that utilize similar approaches to address homelessness are ways to put learned concepts and skills into action for better patient care.

### Structural racism

Housing inequity is a form and mechanism of structural racism and, as such, has implications for adverse health outcomes. Structural racism refers to societal racial discrimination through systems of housing, education, employment, earnings, benefits, credit, media, health care, and criminal justice.^52^ Inequitable housing opportunity, through historical practices such as redlining, has manifested in poor health outcomes for racial minorities that still exist in EDs today. The term redlining refers to a previous legal practice developed in the 1930s by the Federal Housing Administration that marked maps with red lines to delineate neighborhoods where mortgages were denied to marginalized groups.^53^ Bailey et al.^53^ further described how this process of racial residential segregation has resulted in many ways in which housing injustice and insecurity negatively affects health, including the substandard quality of living conditions, exposure to pollutants and toxins, and restricted access to quality health care.

The health effects of inequitable housing and poor living conditions are apparent in the manifestation of asthma in the ED. An ecological study of eight California cities reported that historically redlined non‐Hispanic Black and Hispanic neighborhoods were associated with a relative risk of 1.39 in rates of ED visits due to asthma compared with that of the lowest‐risk neighborhoods.^54^ In September 2020, Bryant‐Stephens et al.^55^ described asthma as critically related to housing and highlighted multiple studies linking indoor aeroallergens and living conditions to health outcomes. Substandard housing conditions and exposure to pollution creates a living environment full of potential asthma triggers, such as mold, cockroach, mouse, and dust mite allergens.^56^ Early childhood exposure to these allergens results in increased risk of asthma,^57^ while continued exposure may exacerbate asthma symptoms and increase ongoing risk of asthma morbidity.^57^, ^58^

Understanding the context of patient risk factors and health outcomes associated with structural racism and poor living conditions is essential to the practice of EM and resident preparedness. Programs can engage with community leaders who advocate for racial justice and invite them to speak at residency conference to hear firsthand perspectives on the structural impact of racism. Residents can also visit community centers that primarily serve racial minorities to gain better insight into how public policy and the built environment affects health. Bailey et al.^53^ mentioned the historical influence of structural racism on the teaching of health sciences and offered a promising outlook that today's medical and public health students, motivated by the Black Lives Matter movement, have advocated for the incorporation of racism and health into standard coursework. EM residency curricula should continue to develop this approach to education and challenge residents to make everyday connections between race, housing, and health.

## IMPLICATIONS FOR EDUCATION AND TRAINING IN EM

For homelessness to be prioritized as a common and significant SDoH that is integral to the training and practice of EM, important future steps include the development and sharing of formal EM residency curricula dedicated to this topic. As demonstrated by the needs assessment, formal teaching on homelessness had a small presence in EM education; however, it was overall recognized as important and desired to be a larger component of curricula. The largest barriers to accomplishing this were time and content expertise among faculty. Our discussion of four key topics indicates that homelessness has a significant impact on health and presents many educational needs that can be addressed through various teaching methods. To this end, a more intentional emphasis on education and training is not only possible but needed. If formal curricula are to thrive, content should be made accessible through sharing on platforms such as literature publications, EM annual assemblies, and free open‐access medical education (FOAMed).

Prioritizing homelessness in EM education has certain speculative benefits. The ED is uniquely positioned to encounter the health impacts of homelessness better than any other specialty. Improving education and training on this topic presents an opportunity for EM to own the expertise necessary to address homelessness. Better education and awareness can prompt physicians to make screening for housing status routine, with likely positive effects on patient care, documentation, and use of social resources. Robust screening can lead to an emphasis on including homelessness in diagnosis coding, resulting in more accurate identification of individuals at risk of adverse health outcomes. Continued research and quality improvement efforts to further explore these connections are essential for progress. Introducing structured learning on this topic could improve the overall knowledge, clinical skills, and wellness of residents. In recent years, wellness has become a growing priority within EM. Better preparing residents to effectively care for this patient population may lead to less burnout, increased job satisfaction, and more overall success in residency.

While EM may not fix societal inequities, they are common in the ED and should be understood by emergency physicians. Evidence has demonstrated the appreciable impact of housing on different aspects of health. Through the development of education and training on these issues in the care of individual patients, physicians can have agency to improve health in meaningful ways. Prioritizing the intersection of homelessness, housing, and health in EM residency curricula is a continued step forward toward better physician training and complete care of patients in the ED.

## Supporting information

Supplementary Material
