# Supplementary material for: Prioritizing homelessness in emergency medicine education: A concept paper
Source: AEM Educ Train. 2022 Jun 23;6(Suppl 1):S85–92. doi: 10.1002/aet2.10753 (PMC9222893; doi:10.1002/aet2.10753)
Supplement: Supplementary file 1 — Supplementary Material [file AET2-6--s001.docx]

APPENDIX 1

EM Residency Needs Assessment Survey

1. Please indicate what state your program is located in:
2. Please indicate the length of your program:
   1. 3 years
   2. 4 years
3. Has your program’s education curriculum been active for at least 1 year?
   1. Yes
   2. No
4. Your program’s primary setting can best be described as: [select one]
   1. Urban
   2. Suburban
   3. Rural
5. Your program’s primary rotation site(s) can best be described as (select all that apply):
   1. Academic
   2. County
   3. Private/Community
   4. Military
6. How would you describe your residents’ exposure to patients experiencing housing insecurity? [select one]
   1. Nonexistent
   2. Minimal
   3. Moderate
   4. Somewhat Heavy
   5. Heavy
7. How would you describe your program’s priority of including homelessness education in your curriculum? [select one]
   1. Not a priority
   2. Somewhat of a priority
   3. Mostly a priority
   4. High priority
8. How would you describe your program’s priority of including social determinants of health in your curriculum and patient care? [select one]
   1. Not a priority
   2. Somewhat of a priority
   3. Mostly a priority
   4. High priority
9. Does your curriculum include any of the following mandatory elements that specifically address housing insecurity (select all that apply):
   1. Dedicated didactics/lectures
   2. Incorporation of topic into medical lectures/cases
   3. Workshop/Small groups
   4. Journal club
   5. Collaboration with Social Work
   6. Simulations
   7. Immersion experiences
   8. None of the above
   9. Other:
10. Does your program offer any of the following optional activities that address housing insecurity (select all that apply):
    1. Social EM or housing insecurity elective
    2. Free clinic or street medicine clinical opportunities
    3. Community organization partnerships
    4. None of the above
    5. Other:
11. During each year of your curriculum, approximately how many hours on average are dedicated to specifically addressing housing insecurity?
12. In your opinion, during each year of curriculum, approximately how many hours on average should be dedicated to specifically addressing housing insecurity?
13. What do you perceive as barriers to implementing or improving homelessness education in your curriculum? (select all that apply)
    1. Limited availability of faculty for this topic
    2. Limited content expertise by faculty for this topic
    3. Lack of time available in the curriculum
    4. Lack of funding
    5. Lack of interest by program leadership
    6. Lack of interest by residents
    7. Not enough patients experiencing homelessness to consider it a priority
    8. No barriers
    9. Other:
14. Please indicate the importance of the following educational elements as they pertain to homelessness, even if they are not currently part of your curriculum: [scale of 1 to 5, with 1 = no value and 5 = highest value]
    1. ACGME competency-based educational goals
    2. Didactics/lectures
    3. Incorporation of topic into medical lectures/cases
    4. Workshop/Small groups
    5. Journal club
    6. Collaboration with Social Work
    7. Simulations
    8. Immersion experiences
    9. Community organization partnerships

Please feel free to add any comments about homelessness/housing insecurity in EM education:
